# Care strategies for people with cancer in light of Uncertainty in Illness Theory

**DOI:** 10.1590/0034-7167-2025-0491

**Published:** 2026-07-31

**Authors:** Júlia Mesko Silveira, Lílian Moura de Lima Spagnolo, Rosiane Filipin Rangel, Franciele Roberta Cordeiro, Eda Schwartz, Thalison Borges de Oliveira, Mariléia Stübe, Carolina Badin de Oliveira

**Affiliations:** I Universidade Federal de Pelotas. Pelotas, Rio Grande do Sul, Brazil; II Universidade Federal do Rio Grande. Rio Grande, Rio Grande do Sul, Brazil

**Keywords:** Adaptation, Psychological, Coping Skills, Uncertainty, Neoplasms, Patients., Adaptación Psicológica, Habilidades de Afrontamiento, Incertidumbre, Neoplasias, Pacientes.

## Abstract

**Objectives:**

to describe the care strategies used by people with cancer in the face of uncertainty in illness.

**Methods:**

a qualitative study was conducted at an oncology unit in southern Brazil, using semi-structured interviews with 14 people undergoing cancer treatment in July 2024, which were subsequently subjected to categorical content analysis.

**Results:**

it was identified that people undergoing cancer treatment use different care strategies to cope with uncertainty in illness, including artistic activities, psychological support, use of technology, maintenance of daily activities, attention to appearance, leisure time and celebration with family, connection with the environment, and complementary therapies.

**Final Considerations:**

the strategies mentioned positively impacted coping with uncertainty and strengthening the autonomy of people undergoing cancer treatment.

## RESUMEN

**Objetivos:**

describir las estrategias de atención empleadas por personas con cáncer ante la incertidumbre de la enfermedad.

**Métodos:**

se realizó un estudio cualitativo en una unidad de oncología del sur de Brasil, mediante entrevistas semiestructuradas con 14 personas en tratamiento oncológico en julio de 2024, que posteriormente se sometieron a un análisis de contenido categórico.

**Resultados:**

se identificó que las personas en tratamiento oncológico utilizan diferentes estrategias de cuidado para afrontar la incertidumbre de la enfermedad, incluyendo actividades artísticas, apoyo psicológico, uso de tecnología, mantenimiento de las actividades cotidianas, cuidado de la apariencia, tiempo libre y celebraciones familiares, conexión con el entorno y terapias complementarias.

**Consideraciones Finales:**

las estrategias mencionadas tuvieron un impacto positivo en el afrontamiento de la incertidumbre y el fortalecimiento de la autonomía de las personas en tratamiento oncológico.

## INTRODUCTION

Cancer is a chronic non-communicable disease that impacts not only the body but also individuals’ emotional and psychological status. Feelings such as fear, anxiety, and uncertainty about the future become common, adding to concerns related to self-image and the social role a person plays. Therefore, the importance of emotional support from diagnosis to treatment and recovery is highlighted, contributing to adaptation^(1)^.

In 2021, Brazil established the Statute of Persons with Cancer, which aims to guarantee the promotion of equitable conditions in the different phases of the disease, such as the right to early diagnosis through care offered by the Brazilian Health System, access to quality treatments and disease prevention, as well as the mandatory dissemination of transparent information about the disease and its treatment^(2)^. However, research^(3,4)^ shows that there are delays in diagnosis and treatment provided by health systems to people with cancer. In this context, a study carried out in Ceará identified delays in test results, ineffectiveness of professionals in investigating clinical manifestations, issues related to transportation and food, as well as the need for psychosocial support^(5)^.

In this scenario experienced by a person with cancer, the applicability of uncertainty in Illness Theory proposed by Merle Mishel stands out^(6,7)^. This theory falls within the middle-range theories, which arise as a response to the need to understand observed patterns in behavior, organization and social changes, without seeking a comprehensive explanation in a single theory^(8)^.

The aforementioned theory defines uncertainty in illness as a mental state resulting from insufficient information to form a cognitive schema or assign meaning to a situation or event. Uncertainty may not be resolved, but it can possibly be transformed, through coping strategies, from danger into opportunity, implying a positive outcome^(6,7)^.

Given the above, the potential of this research becomes apparent when viewed through the lens of Uncertainty in Illness Theory regarding individuals with cancer. A cancer diagnosis brings a series of challenges and uncertainties, resulting in multidimensional disorder, as a person is confronted with questions about the meaning of life and terminal illness, thus giving rise to a new order. This process is dynamic and influenced by the ecosystem in which human beings are embedded and the care strategies adopted throughout the course of the illness and treatment^(9)^.

A systematic literature review, conducted between November and September 2023 in the MEDLINE/PubMed, BDENF, and LILACS databases, identified 15 studies on care strategies used by people with cancer published between 2002 and 2020. It was observed that spirituality, religion, and religiosity are the most recurrent forms of coping^(10,11)^. However, a scarcity of research addressing other dimensions of care was observed, such as daily, relational, and subjective practices, which also influence how individuals cope with the uncertainty and suffering arising from illness. Furthermore, it was found that less than half of studies were produced by nurses, indicating a gap in nursing’s contribution to this field of knowledge. Thus, the present study was based on the theoretical and practical need to broaden the understanding of the multiple care strategies adopted by people with cancer, contributing to strengthening the nursing perspective on self-care and coping with uncertainty in the oncological context.

Given the above, the guiding question was formulated: what care strategies are used by people with cancer in the face of uncertainty in illness?

## OBJECTIVES

To describe the care strategies used by people with cancer in the face of uncertainty in illness.

## METHODS

### Ethical aspects

The research was approved by the Research Ethics Committee of a public university in Brazil, and was in accordance with Resolution 466/2012^(12)^ for research involving human beings.

To ensure anonymity, participants were identified by the letter “I”, followed by the letters “F” (referring to female) and “M” (referring to male), in addition to the interview number.

### Theoretical-methodological framework

This study was based on uncertainty in Uncertainty in Illness Theory, proposed by Merle Mishel^(6,7)^, which provided theoretical support for data analysis and results construction. From this perspective, the aim was to understand participants’ experience in the face of uncertainty associated with the disease, articulating the data with the three central concepts of the theory: antecedents of uncertainty; uncertainty assessment; and strategies for coping with uncertainty.

### Study design

This is qualitative research. The rigor and quality criteria analyzed in the research were carried out in accordance with COnsolidated criteria for REporting Qualitative research.

### Study setting

The research was conducted in a municipality in Rio Grande do Sul, in an oncology unit of a teaching hospital affiliated with *Empresa Brasileira de Serviços Hospitalares*.

### Data source

The study was conducted with people undergoing cancer treatment (chemotherapy and radiotherapy). Individuals with cancer who were receiving treatment at healthcare facilities, aged 18 or older, able to communicate verbally in Portuguese, and aware of their cancer diagnosis were selected. Those with clinical conditions that prevented them from answering the questions were excluded.

Eighteen potential participants were identified; of these, four were excluded: two for personal reasons and two due to the pilot test. Thus, through purposive sampling, a total of 14 participants were selected. Data saturation was reached when the collected information proved sufficient to answer the objectives, with no new relevant elements emerging^(13)^.

The individual semi-structured interviews, conducted with people undergoing cancer treatment, were audio-recorded and transcribed in full, constituting the main source of data for analysis. The *corpus* consisted of textual transcriptions, which fully preserved the speech content. No field notes or other supplementary records were used.

### Data collection and organization

Data collection took place after the research project was approved by the Research Ethics Committee in July 2024. The first author of the article made in-person contact with the physician responsible for the unit, informing them of the study’s objective and method. The research was also presented to the nursing staff, who were asked to nominate participants according to the study’s inclusion criteria. A room was made available for data collection, as well as access to the list of people undergoing treatment in the unit and to medical records to confirm that potential participants were aware of their diagnosis.

After a preliminary selection from the daily list, potential participants were approached at the service reception while they waited for their appointment. Upon acceptance, the interview was scheduled according to participants’ availability at a location of their preference, prioritizing the use of a room within the service itself. Of the 14 participants, only one chose to have the interview at home. The others had the interview in the service room.

Data were collected through semi-structured interviews with questions about participants’ profiles and their experience with cancer. The interviews were audio-recorded and subsequently transcribed manually in full by two previously trained students, resulting in 71 pages. The mean interview time was 23 minutes and 43 seconds.

### Data analysis

The data underwent categorical content analysis. This technique allows for the analysis of verbal and nonverbal communication recorded during the interviews, and is systematized in three stages: pre-analysis; material exploration; and treatment of results and interpretation^(14)^.

In the pre-analysis phase, which involved organizing the materials, a preliminary reading of the collected data was conducted, along with the hypothesis formulation and the *corpus* definition. Following this, in material exploration, the processes of data coding, categorization, and enumeration were applied. Finally, the last stage consisted of processing and interpreting the results, allowing for the formulation of inferences and the analysis of the objectives and hypotheses established based on the collected data^(14)^.

For the analysis of results, a directed analysis was used, according to pre-established categories of Uncertainty in Illness Theory concepts. In this process, concepts are used as guides for the initial codes of content analysis. The codes are defined before and during data analysis, and are derived from theory or from relevant preceding research results^(15)^.

Thus, a folder was created in Google Drive^®^ to store the audio recordings and the compiled texts of the transcripts. Subsequently, data skimming was performed, manually assigning a color to each of the three main concepts of Uncertainty in Illness Theory^(9)^: antecedents of uncertainty; strategies for dealing with uncertainty; and adaptation. Subsequently, the speeches were grouped according to the concepts and organized in another document within the same folder. Chart 1 shows the details of data coding and categorization operationalization.

**Chart 1:** Example of data coding and categorization operationalization

Theoretical concept	Empirical category	Recording unit (Speech example)
Antecedents of uncertainty	Knowledge about cancer and the ability to process diagnosis and treatment in the face of uncertainty in illness	*I try not to know anything. I prefer to let things roll like this, and everything’s fine.* (IF2) *The ground opened up, there was fear, there was uncertainty, there was panic, there was everything at the same time.* (IF11)
Strategies for dealing with uncertainty	Coping: care strategies and resources used to address uncertainty in illness	*Drawing, writing, I write and I draw. So, doing these things helps me a lot. Singing is something that is part of my profession, and it also helps me a lot, it’s like it’s therapeutic.* (IF1) *Therapy with the psychologist is a form of self-care that I also have now* [...] *things come up and you understand, you understand that you need it and it’s doing me a lot of good, I’m enjoying it.* (IF10)
Adaptation	Self-organization processes and probabilistic thinking	*I get up, take my shower, go have lunch with my daughter. I collect laundry, I help hang the clothes on the line, I fold the clothes for her. I get dressed too.* (IF6) *I am undergoing treatment and I will get better. Many times, I’m left with this doubt, “Will I be able to do it? Will I not?”* [...] *I am more confident; everything will be alright. It’s just a bad phase.* (IF3)

Three categories emerged from this, and this work will present the category that addresses coping strategies, a term from Merle Mishel’s Uncertainty in Illness Theory. This term is conceptualized as the set of cognitive and behavioral strategies used by individuals to deal with situations perceived as dangerous. In contexts of uncertainty, coping involves efforts directed at reducing uncertainty and managing the emotions associated with the perception of danger^(9)^.

## RESULTS

Among the 14 participants, the age ranged from 21 to 72 years. The sex was predominantly female 85.7% (12), and 64.3% (9) had children. Concerning marital status, 42.8% (6) were married. In relation to occupation, 71.4% (10) were not working. Regarding religion, 35.7% (5) declared themselves *Umbandists*. Breast cancer predominated as a diagnosis with 42.9% (6), and 42.9% (6) underwent chemotherapy, with treatment time ranging from one month to seven years, with an average of two years.

### Care strategies in the face of uncertainty in illness and factors that influence it

The care strategies adopted by participants revealed distinct ways of coping with uncertainty inherent in cancer illness and treatment, combining social and self-care practices. Art emerged as an ally in facing diagnosis and treatment. It is used as therapy to express emotions and give new meaning to the experience of the disease:

*Drawing, writing, I write and I draw. So, doing these things helps me a lot. Singing is something that’s part of my profession, and it also helps me a lot, it’s like therapy.* (IF1)

*I went to the CRAS, because since I was alone at home, and I have a lot of paint, I really like to paint. I’m painting a canvas that’s 1 by 1.5 meters and a headboard. Sewing too, a lot of sewing.* (IF10)

Seeking emotional support, such as therapy with a psychologist, allowed for a better understanding of the illness and individual needs. It also became evident that the use of mobile devices is a form of coping and distraction. This process facilitates acceptance of the illness and internal reorganization, contributing to the search for meaning amidst uncertainty.

*Therapy with a psychologist is also something I’m taking care of now* [...] *things come to me and you start to understand. You understand that you need it and it’s doing me a lot of good, I’m enjoying it.* (IF10)

*I’m on my phone, I browse Facebook^®^ to see who has a birthday. I do everything on my phone.* (IF12)

Maintaining daily activities and taking care of one’s appearance emerged as ways to reaffirm personal identity and minimize the emotional impact of the disease. They reflect an effort by interviewees to preserve a sense of normalcy and control in the face of the uncertainties caused by cancer.

*I go to work, I go to the gym, I drink socially. Like, I go out, I walk, I’m very vain. I always get my gel nails and eyelashes done, even now.* (IF2)

*I didn’t really like looking at myself in the mirror before, and now what I do most is look at myself in the mirror. Every day I look in the mirror and every day I’m grateful for another day I’m here.* (IF11)

The search for moments of leisure and celebration with family emerged as a significant strategy for coping with the uncertainty generated by the diagnosis, proving to be a mechanism for regaining normalcy, maintaining identity, and reaffirming life.

*After the diagnosis, I started that fight! Since my birthday was at the end of the month, I didn’t tell anyone, I kept it to myself. Then I waited until after my birthday* [...] *I arranged to have a barbecue to get the whole family together for my birthday. I said, “Let’s go camping, that’s what we like”, and so I did! I want cake, I want birthday wishes, I want everything! And that’s what I wanted, I wanted to feel good, I wanted to feel alive, you know?* (IF9)

*I like to go out with my husband and take walks; I love going to a bar. We like to have a non-alcoholic beer.* (IF14)

Contact with nature was also mentioned as a strategy for dealing with negative feelings and finding inner strength. These practices reflect the importance of this coping strategy in the face of experienced uncertainty.

[...] *I really enjoy going into the woods. There I feel good and I start talking to you, and then I cry a lot. When it’s over, I go back home feeling cleansed of everything. I like having my time alone, it’s where I renew myself.* (IF11)

[...] *my husband and I go out a lot, which is something we really enjoy. And I walk in those fields, by those waterfalls, and sitting there, that makes me feel so good.* (IF14)

The search for complementary therapies was also highlighted by participants as a care strategy. Furthermore, one participant indicated the use of spiritual surgery linked to religion as a way to cope with uncertainty.

*I drink teas, teas to boost immunity, I drink chamomile tea, aranto tea, fennel tea.* (IF11)

*The care strategy I had was through religion, I had spiritual surgery. I do applications, there’s Mother Jurema who says it’s surgery.* (IF12)

### Resources accessed by people with cancer in the face of uncertainty in illness

Following a cancer diagnosis, the resources accessed play a fundamental role in reorganizing uncertainty. In this research, there was mention of healthcare professionals, the support network composed of family, friends, and neighbors, as well as religion and faith.

The statements highlight physicians as central figures in mediating uncertainty through diagnosis and the provision of information.

*And it was the physicians themselves who explained it to me from the beginning. As soon as I started to suspect it, the physicians started telling me what it was.* (IF1)

[...] *the physician spoke directly, you know. From the exam I had, it was already clear what it was, but just knowing and hearing it from the physician isn’t easy, because my cancer, the physician isn’t quite sure, but it’s in my spine.* (IF3)

Another resource mentioned was family support, one of the most frequently accessed to cope with a cancer diagnosis and treatment. Sources of support include family, friends, and neighbors, reinforcing the collective role of the support network and playing a crucial part in reorganizing oneself amidst uncertainties.

*I have a very close friend, who is also my godmother, so she’s the one I can confide in. So, in the end, it would be my husband and my friend. And being with my son, my son who gives me the strength to go on.* (IF3)

*Family is everything. I have a son who lives next door, who takes care of me the most because I live there. My daughter-in-law, I believe she is my guardian angel, my protector, because I said: “Thanks to her I’m here today, because she made everything easier”.* (IF12)

Finally, participants’ reports revealed the influence of religion, especially *Umbanda*, in coping with cancer. The religious community emerged as a source of emotional, practical, and spiritual support, helping them deal with the challenges of treatment and find the strength to move forward, emerging as a refuge amidst uncertainty.

*There* [Umbanda religion] *is where I get my energy. It’s by participating, you know, on the days they hold services, receiving a word from a fellow member, from a spiritual guide, that all this will pass, that I’m not alone, that there is a God, there is a healer who is our father Oxalá so that’s where I also get some energy for myself.* (IF8)

*The evangelical religion is very important. I’m not an active attendee, you know, like, a regular one. I really like going on Sundays, but it did me a world of good. It made all the difference; it helped me a lot to face things from the beginning. My family was very important, but the church itself helps you much more if you have faith, you know; it made all the difference.* (IF9)

Faith in a higher power provided a perspective of hope and a sense of purpose, alleviating the anguish and uncertainty in the face of a cancer diagnosis:

*I have a lot of faith, a lot of faith, a lot of belief. God is my foundation.* (IF10)

*I think that if God didn’t exist in my life, if Christ didn’t exist in my life, I don’t know what I would have gone through. Since the day of my surgery, after treatment, I’ve always been certain. In the most difficult moments, God carried me in His arms, and in others, He always extended His hand to me.* (IF11)

## DISCUSSION

Cancer, by posing significant challenges due to its chronic and potentially lethal nature, awakens uncertainties in people, especially regarding prognosis and treatment effectiveness. These uncertainties generate adverse feelings that can emotionally destabilize individuals. In this context, understanding the care strategies adopted by people with cancer in the face of uncertainty in illness becomes essential, considering the emotional and social complexity associated with this condition^(7,9,16)^.

Care strategies in coping with cancer constitute forms of coping, i.e., cognitive and behavioral strategies used to restore balance in the face of unpredictable situations^(6)^. In this study, the use of art during treatment was identified, playing a fundamental role in managing the uncertainty that permeates this moment, providing people with a way to express feelings and process emotions^(7,9)^. Furthermore, these artistic practices allow for a reinterpretation of the lived experience, transforming the experience of diagnosis and treatment into something that can be understood and faced in a more positive way.

A study conducted with adults with onco-hematological diseases at a public university hospital in Goiás highlighted that art therapy is an effective form of personal expression, contributing to the strengthening of individual capacities, coping with the disease, and accessing deep emotions^(17)^. Art therapy can reduce anxiety and depression, and improve quality of life, making it an effective therapeutic approach in the care of people with cancer^(18)^.

Psychological therapy stood out as a coping strategy by promoting acceptance of the disease, emotional reorganization, and the search for meaning amidst uncertainty. This finding converges with research^(19)^ that argues that psychological therapeutic support is as essential as other cancer treatments. This is because psychological interventions offer emotional support and help cope with the challenges of the disease and treatment, enabling individuals to deal with the emotional challenges and repercussions of cancer, while also addressing existential questions about living and dying, promoting adaptive and humanized coping^(19)^.

The use of mobile devices has also been referred to as a coping strategy and a means of distraction and support during treatment. A study carried out in Maringá^(20)^ showed that access to qualified information through these technologies promotes self-care, empowerment and autonomy in decision-making, reducing uncertainties and strengthening protective factors. Thus, smartphone-based interventions prove effective for the development of skills related to self-care and shared decision-making.

Both emotional support strategies and the use of mobile devices play complementary roles in coping with cancer. Psychological therapy is essential for the support and internal reorganization of people with the disease. On the other hand, mobile devices, when used as educational and distraction tools, promote self-care, strengthen autonomy, and reduce the uncertainties faced during treatment^(7,9)^.

Some participants in this study adopted self-care strategies to cope with the uncertainties caused by cancer, such as maintaining daily routines and taking care of their appearance. These actions reflect an effort to preserve a sense of normalcy, strengthen self-esteem, and face the disease with more control over their bodies and lives. Similarly, a study^(21)^ showed that the side effects of cancer treatment can negatively impact women’s quality of life. Regarding body image, many participants reported not feeling attractive or satisfied with their appearance, especially in cases of breast and cervical cancer. This is linked to stereotypes that sexualize and value specific parts of the female body, such as breasts and hair, making the physical changes caused by treatment a source of dissatisfaction. These aspects reinforce the need for healthcare professionals to address sexuality issues in consultations, considering their central role in self-esteem.

A participant, in particular, reported a significant transformation in her relationship with her body and with life, demonstrating a process of emotional adaptation in the face of the uncertainties caused by cancer. The change in how she began to see herself in the mirror reflects a reinterpretation of her own body, promoting a new perception of gratitude and acceptance in the face of the vulnerability imposed by the disease. Similarly, research^(22)^ with women with breast cancer after mastectomy has shown that body image is shaped throughout life by experiences, transitions and significant events, such as illnesses that affect the relationship with the body and the environment. In cancer treatment, physical, emotional and social changes can alter self-image and negatively impact the experience of sexuality. However, after the treatment process and its completion, beauty takes on a new meaning, valuing the body reconstructed from the inside out. Overcoming cancer comes to symbolize the strength and victory of a woman who fought and won, challenging the image of fragility associated with the disease and reinterpreting it as a symbol of beauty and achievement.

These strategies highlight the role of self-care and maintaining daily habits in coping with the illness, allowing both individuals to maintain their identity and a sense of control over their lives amidst the challenging context they are experiencing. These experiences not only identify a process of emotional adaptation but also an attempt to deal with the uncertainties caused by the illness.

As identified in this study, the pursuit of leisure time, family time, and contemplation of nature becomes a way to regain control and a sense of normalcy after diagnosis. The search for emotional well-being, happiness, and connection, even in the face of uncertainty in illness, appears as a strategy to cope with negative feelings and find inner strength^(7,9)^.

Similar findings were identified in research^(23)^ in the field of oncology, which revealed diverse meanings for leisure. Among the activities most cited as representative of leisure are walks, trips, family gatherings and appreciation of nature. Leisure, therefore, has an individualized meaning, varying from person to person. These practices are directly linked to the satisfaction of personal needs and, above all, to the development of skills and the increase in well-being of each person. These practices reflect the importance of this approach as a way of coping with the situation experienced.

The search for complementary therapies, identified among the participants of this research, aimed to care for the body and control the adverse effects of treatments, including the use of medicinal plants and adherence to religious practices. These approaches demonstrate how religiosity offers comfort and hope, helping to cope with the uncertainties of the illness process^(7,9)^.

In this regard, a study on care practices carried out with participants living with cancer in the city of Pelotas stands out. It highlighted that the biomedical model, by itself, does not meet all people’s needs, as each individual attributes different meanings to the experience of illness. Therefore, many resort to practices beyond conventional treatment, such as the use of teas, dietary changes, folk remedies, Reiki, folk medicine, and even astral surgery, in search of relief or a cure. Care approaches varied according to the needs of each participant and, although unconventional, were perceived as effective. Thus, these complementary therapies can be adopted in conjunction with traditional treatment, aiming to minimize side effects and improve quality of life^(24)^. The integration of complementary therapies with traditional medicine is seen as a way to deal with the uncertainties associated with the process of illness and recovery^(7,9)^.

Given the complexity of the care required by individuals with cancer, it is essential to ensure continuity of care beyond the hospital or outpatient setting, allowing them to feel supported and safe to face all phases of treatment. Among the professionals in the multidisciplinary healthcare team, nursing plays a fundamental role in supporting the coping with cancer, employing strategies aimed at minimizing physical and emotional discomfort, providing a better quality of life^(25)^.

In line with the above, a study on the role of nurses in oncology care indicates that nurses are recognized for their ability to integrate the healthcare team, patients, and their families. Through assertive and objective communication, they are able to guide, clarify, reinforce, and validate information received from different sources. The empowerment provided by healthcare professionals to people with cancer, through adequate information, fosters self-awareness and active participation in treatment planning, resulting in greater self-confidence and improved quality of life^(26)^.

Understanding these processes of uncertainty and coping is essential for nursing, which occupies a strategic position in supporting the adaptation of people with cancer. Nursing care should, therefore, incorporate approaches that favor the construction of meaning and the strengthening of coping strategies, such as those evidenced in this research. Given this, it becomes essential to implement these care strategies in nursing care in chemotherapy and radiotherapy outpatient clinics. Nurses play a central role in humanization of care, ensuring comprehensive and individualized support.

### Study limitations

Limitations of this study include the fact that only one institution was the research setting, which necessitates a review of results. Furthermore, the initial interviews required an adaptation to a qualitative approach, particularly in reformulating the questions, to more effectively capture participants’ subjective and experiential elements.

### Contributions to nursing, health, or public policy

This research contributes to the field of knowledge by broadening the understanding of the experiences of people with cancer facing uncertainty in illness, offering support for the improvement of care practices. The findings can underpin actions aimed at implementing these care strategies in nursing care that help people with uncertainty in illness, reinforcing the importance of humanized and person-centered care in the oncological context. Furthermore, the results have direct implications for training nursing professionals, providing tools and strategies that can be incorporated into continuing and permanent education, aiming at the development of competencies in humanized care, therapeutic communication, and addressing uncertainty. In the research context, they stimulate future investigations into interventions that enhance patient-centeredness and evidence-based practice. Finally, they reinforce the importance of integrating these strategies into clinical practice, promoting safer, more effective care centered on the individual needs of people with cancer.

## FINAL CONSIDERATIONS

This research revealed that people with cancer adopt, as coping strategies to deal with uncertainty in illness, artistic practices, psychological support, use of technology, maintaining daily activities, attention to appearance, leisure time and celebration with family, contact with nature, and complementary therapies. These strategies demonstrate the self-organizational capacity and probabilistic thinking of a person affected by cancer, highlighting the positive impact of these practices in coping with uncertainty and strengthening their autonomy. Among the resources accessed by participants were healthcare professionals, the family and friends support network, as well as faith and religion. Regarding religious practices, *Umbanda* was the most frequently mentioned, standing out as a source of support and comfort.

It is suggested that future research investigate adherence to these strategies from the beginning of treatment, monitoring the progress of people with cancer who do not use such strategies and comparing them with those who implement them over time.

## Data Availability

The research data are available within the article.
