# Supplementary material for: Care strategies for people with cancer in light of Uncertainty in Illness Theory
Source: Rev Bras Enferm. 2026 Jul 31;79:e20250491. doi: 10.1590/0034-7167-2025-0491 (PMC13435856; doi:10.1590/0034-7167-2025-0491)
Supplement: Translation

# Translation: Estratégias de cuidado de pessoas com câncer à luz da Teoria da Incerteza da Doença

**DOI:** 10.1590/0034-7167-2025-0491pt

**Authors:** Júlia Mesko Silveira, Lílian Moura de Lima Spagnolo, Rosiane Filipin Rangel, Franciele Roberta Cordeiro, Eda Schwartz, Thalison Borges de Oliveira, Mariléia Stübe, Carolina Badin de Oliveira

**Affiliations:** I Universidade Federal de Pelotas. Pelotas, Rio Grande do Sul, Brasil; II Universidade Federal do Rio Grande. Rio Grande, Rio Grande do Sul, Brasil

**Keywords:** Adaptação Psicológica, Habilidades de Enfrentamento, Incerteza, Neoplasias, Pacientes.

## Abstract

**Objetivos:**

descrever as estratégias de cuidado utilizadas por pessoas com câncer diante da incerteza da doença.

**Métodos:**

estudo qualitativo, realizado em unidade de oncologia no sul do Brasil, aplicando-se entrevistas semiestruturadas a 14 pessoas em tratamento oncológico, em julho de 2024, posteriormente submetendo-as à análise de conteúdo, modalidade categorial.

**Resultados:**

identificou-se que as pessoas em tratamento oncológico utilizam diferentes estratégias de cuidado para lidar com a incerteza da doença, abrangendo atividades artísticas, acompanhamento psicológico, uso de tecnologia, manutenção das atividades cotidianas, cuidado com a aparência, momentos de lazer e celebração com a família, conexão com o meio ambiente, e terapias complementares.

**Considerações Finais:**

as estratégias referidas impactaram positivamente o enfrentamento da incerteza e o fortalecimento da autonomia da pessoa em tratamento oncológico.

## INTRODUÇÃO

O câncer é uma doença crônica não transmissível que impacta não apenas o corpo, mas também o estado emocional e psicológico do indivíduo. Sentimentos como medo, ansiedade e incerteza quanto ao futuro se tornam comuns, somando-se às preocupações relacionadas à autoimagem e ao papel social que a pessoa desempenha. Diante disso, destaca-se a importância do suporte emocional desde o diagnóstico até o tratamento e recuperação, contribuindo para a adaptação^(1)^.

Em 2021, no Brasil, foi estabelecido o Estatuto da Pessoa com Câncer, que tem como propósito garantir a promoção das condições de equidade nas diferentes fases da doença, como o direito ao diagnóstico precoce por meio dos cuidados oferecidos pelo Sistema Único de Saúde, o acesso a tratamentos de qualidade e a prevenção da enfermidade, além da divulgação obrigatória de informações transparentes sobre a doença e o tratamento^(2)^. No entanto, pesquisas^(3,4)^ demonstram que há demora no diagnóstico e tratamento fornecidos pelos sistemas de saúde às pessoas com câncer. Nesse contexto, estudo realizado no Ceará identificou morosidade nos resultados de exames, ineficácia dos profissionais na investigação das manifestações clínicas, questões relacionadas ao transporte e à alimentação, além da necessidade de suporte psicossocial^(5)^.

Nesse panorama vivenciado pela pessoa com câncer, destaca-se a aplicabilidade da Teoria da Incerteza da Doença proposta por Merle Mishel^(6,7)^. Tal teoria se enquadra dentro das teorias de médio alcance, as quais surgem como resposta à necessidade de compreender padrões observados em comportamento, organização e mudanças sociais, sem buscar uma explicação abrangente em uma única teoria^(8)^.

A teoria supracitada define a incerteza na enfermidade como um estado mental decorrente de informações insuficientes para formar um esquema cognitivo ou atribuir significado a uma situação ou evento. A incerteza pode não ser resolvida, mas possivelmente se transforma, a partir de estratégias de enfrentamento, de perigo em oportunidade, implicando um resultado positivo^(6,7)^.

Diante do exposto, vislumbra-se a potencialidade desta pesquisa com o olhar da Teoria da Incerteza da Doença sobre a pessoa com câncer. O diagnóstico de câncer traz uma série de desafios e incertezas, os quais resultam em desordem multidimensional, pois a pessoa é confrontada com questões acerca do sentido da vida e da terminalidade, emergindo, assim, uma nova ordem. Esse processo é dinâmico e sofre influências do ecossistema em que o ser humano está inserido e das estratégias de cuidado adotadas no decorrer da doença e do tratamento^(9)^.

Em revisão sistematizada da literatura, realizada entre novembro e setembro de 2023 nas bases MEDLINE/PubMed, BDENF e LILACS, foram identificados 15 estudos sobre estratégias de cuidado utilizadas por pessoas com câncer publicados entre 2002 e 2020. Observou-se que a espiritualidade, a religião e a religiosidade são as formas mais recorrentes de enfrentamento^(10,11)^. No entanto, verificou-se escassez de investigações que abordem outras dimensões do cuidado, como práticas cotidianas, relacionais e subjetivas, que também influenciam a forma como o indivíduo lida com a incerteza e o sofrimento decorrentes da doença. Ademais, constatou-se que menos da metade das produções foi elaborada por enfermeiros, indicando uma lacuna na contribuição da enfermagem para esse campo do conhecimento. Assim, o presente estudo se fundamentou na necessidade teórica e prática de ampliar a compreensão das múltiplas estratégias de cuidado adotadas por pessoas com câncer, contribuindo para o fortalecimento do olhar da enfermagem sobre o autocuidado e o enfrentamento da incerteza no contexto oncológico.

Tendo em vista o exposto, elaborou-se a questão norteadora: quais as estratégias de cuidado utilizadas por pessoas com câncer frente à incerteza da doença?

## OBJETIVOS

Descrever as estratégias de cuidado utilizadas por pessoas com câncer diante da incerteza da doença.

## MÉTODOS

### Aspectos éticos

A pesquisa foi aprovada pelo Comitê de Ética em Pesquisa de uma universidade pública do Brasil, e estava em conformidade com a Resolução n^o^ 466/2012^(12)^ para pesquisas envolvendo seres humanos.

Para garantir o anonimato, os participantes foram identificados pela letra “E”, seguidos das letras “F” (relativo a feminino) e “M” (relativo a masculino), além do número da entrevista.

### Referencial teórico-metodológico

Este estudo se fundamentou na Teoria da Incerteza da Doença, proposta por Merle Mishel^(6,7)^, que ofereceu sustentação teórica para análise dos dados e construção dos resultados. A partir dessa perspectiva, buscou-se compreender a experiência dos participantes diante da incerteza associada à doença, articulando os dados com os três conceitos centrais da teoria: antecedentes da incerteza; avaliação da incerteza; e estratégias para lidar com a incerteza.

### Tipo de estudo

Trata-se de pesquisa qualitativa. Os critérios de rigor e qualidade analisados na pesquisa foram realizados de acordo com o *COnsolidated criteria for REporting Qualitative researc*h.

### Cenário do estudo

A pesquisa foi desenvolvida em um município do Rio Grande do Sul, em uma unidade de oncologia de um hospital escola vinculada à Empresa Brasileira de Serviços Hospitalares.

### Fonte de dados

O estudo foi desenvolvido com pessoas que estavam em tratamento oncológico (quimioterapia e radioterapia). Foram selecionadas pessoas com câncer que estavam realizando tratamento oncológico nos serviços de saúde, com idade igual ou superior a 18 anos, capazes de se comunicar verbalmente em português e cientes do próprio diagnóstico de câncer. Excluíram-se aquelas com condições clínicas que as impossibilitassem de responder aos questionamentos.

Foram identificados 18 potenciais participantes; desses, foram excluídos quatro: dois por motivos pessoais e dois pelo teste piloto. Com isso, mediante amostragem intencional, totalizaram-se 14 participantes. A saturação dos dados foi atingida quando as informações coletadas se mostraram suficientes para responder aos objetivos, não emergindo novos elementos relevantes^(13)^.

As entrevistas semiestruturadas individuais, realizadas com pessoas em tratamento oncológico, foram gravadas em áudio e transcritas na íntegra, constituindo a principal fonte de dados para análise. O *corpus* foi composto pelas transcrições textuais, que preservaram integralmente o conteúdo das falas. Não foram utilizadas notas de campo ou outros registros complementares.

### Coleta e organização dos dados

A coleta de dados ocorreu após aprovação do projeto de pesquisa pelo Comitê de Ética em Pesquisa em julho de 2024. A primeira autora do artigo realizou contato presencial com a médica responsável pela unidade, informando o objetivo e método do estudo. Também foi apresentada a pesquisa para a equipe de enfermagem, sendo solicitada a indicação de participantes de acordo com os critérios de inclusão do estudo. Foi disponibilizada uma sala para a coleta de dados, assim como o acesso à lista de pessoas em tratamento na unidade e aos prontuários para confirmação do conhecimento do diagnóstico por parte do possível participante.

Após a seleção prévia na lista diária, foram abordados os possíveis participantes na recepção dos serviços enquanto aguardavam o atendimento. Diante do aceite, foi realizado o agendamento da entrevista de acordo com a disponibilidade do participante em local de sua preferência, priorizando o uso de sala do próprio serviço. Dos 14 participantes, apenas um escolheu realizar a entrevista em sua casa. Os demais realizaram na sala do serviço.

Os dados foram coletados por meio de entrevista semiestruturada com questionamentos acerca do perfil dos participantes e sobre a sua vivência com a doença oncológica. As entrevistas foram gravadas em áudio e, posteriormente, transcritas manualmente na íntegra por duas discentes previamente treinadas, gerando 71 laudas. O tempo médio das entrevistas foi de 23 minutos e 43 segundos.

### Análise dos dados

Os dados foram submetidos à análise de conteúdo na modalidade categorial. Essa técnica permite analisar a comunicação verbal e não verbal registrada durante as entrevistas, e é sistematizada em três etapas: pré-análise; exploração do material; e tratamento dos resultados e interpretação^(14)^.

Na pré-análise, fase de organização dos materiais, foi realizada uma leitura flutuante dos dados coletados, com a formulação de hipóteses e a definição do *corpus*. Em seguida, na exploração do material, foram aplicados os processos de codificação, categorização e enumeração dos dados. Por fim, a última etapa consistiu no tratamento dos resultados e interpretação, possibilitando a formulação de inferências e a análise dos objetivos e hipóteses estabelecidos a partir dos dados coletados^(14)^.

Para análise dos resultados, foi utilizada a análise direcionada, de acordo com as categorias pré-estabelecidas dos conceitos da Teoria da Incerteza da Doença. Nesse processo, utilizam-se conceitos como guias para os códigos iniciais da análise de conteúdo. Os códigos são definidos antes e durante a análise de dados, e derivam da teoria ou de resultados de pesquisas relevantes precedentes^(15)^.

Assim, foi criada uma pasta no *Google Drive^®^* para o armazenamento das captações de áudio e dos textos compilados das transcrições. Em seguida, fez-se a leitura flutuante dos dados, atribuindo-se manualmente uma cor a cada um dos três principais conceitos da Teoria da Incerteza da Doença^(9)^: antecedentes da incerteza; estratégias para lidar com a incerteza; e adaptação. Posteriormente, as falas foram agrupadas conforme os conceitos e organizados em outro documento dentro da mesma pasta. O Quadro 1 demonstra o detalhamento da operacionalização da codificação e categorização dos dados.

**Quadro 1:** Exemplificação da operacionalização da codificação e categorização dos dados

Conceito teórico	Categoria empírica	Unidade de registro (exemplo de fala)
Antecedentes da incerteza	Conhecimento sobre o câncer e a capacidade de processamento do diagnóstico e tratamento diante da incerteza da doença	*Eu procuro nem saber de nada. Eu prefiro deixar a coisa rolar assim, e tá tudo certo*. (EF2) *O chão se abriu, deu medo, deu incerteza, deu pânico, deu tudo ao mesmo tempo.* (EF11)
Estratégias para lidar com a incerteza	*Coping:* estratégias de cuidado e recursos utilizados no enfrentamento da incerteza da doença	*Desenhar, escrever, que eu escrevo e eu desenho. Então, fazer essas coisas me ajuda bastante. Cantar é uma coisa que está dentro da minha profissão, e também me ajuda muito, é como se fosse terapêutico.* (EF1) *A terapia com a psicóloga é um cuidado que tenho agora também* [...] *as coisas vão vindo e tu vai entendendo, tu entendes que tu precisa e está me fazendo muito bem, estou gostando.* (EF10)
Adaptação	Processos de auto-organização e pensamento probabilístico	*Eu levanto, tomo o meu banho, vou almoçar com a minha filha. Eu recolho roupa, eu ajudo a colocar a roupa na corda, eu dobro a roupa para ela. Visto também.* (EF6) *Eu estou fazendo o tratamento e eu vou me curar. Muitas vezes, fica assim com essa dúvida: “Será que eu vou conseguir? Será que eu não vou?”* [...] *eu estou mais confiante, vai dar tudo certo. É só uma fase ruim.* (EF3)

Emergiram, dessa forma, três categorias, sendo que, neste trabalho, será apresentada a categoria que abordou estratégias de *coping*, termo considerado da Teoria da Incerteza da Doença de Merle Mishel. Este termo é conceituado como o conjunto de estratégias cognitivas e comportamentais utilizadas pelos indivíduos para lidar com situações avaliadas como perigo. Em contextos de incerteza, o *coping* envolve esforços direcionados à redução da incerteza e ao gerenciamento das emoções associadas à percepção de perigo^(9)^.

## RESULTADOS

Entre os 14 participantes, a idade variou de 21 a 72 anos. O sexo foi majoritariamente feminino 85,7% (12), e 64,3% (9) possuíam filhos. Quanto à situação conjugal, 42,8% (6) eram casados. Em relação à ocupação, 71,4% (10) não trabalhavam. No que se refere à religião 35,7% (5) se declararam umbandistas. O câncer de mama predominou como diagnóstico com 42,9% (6), e 42,9% (6) realizaram quimioterapia, com tempo de tratamento variando de um mês a sete anos, com média de dois anos.

### Estratégias de cuidado diante da incerteza da doença e fatores que influenciam-na

As estratégias de cuidado adotadas pelos participantes revelaram formas distintas de lidar com a incerteza inerente ao adoecimento e tratamento do câncer, combinando práticas sociais e de autocuidado. A arte surgiu como uma aliada no enfrentamento do diagnóstico e tratamento. É utilizada como terapêutica para expressar as emoções e ressignificar a experiência da doença:

*Desenhar, escrever, que eu escrevo e eu desenho. Então, fazer essas coisas me ajuda bastante. Cantar é uma coisa que está dentro da minha profissão, e também me ajuda muito, é como se fosse terapêutico.* (EF1)

*Eu procurei o CRAS, que como eu estava só em casa, e eu tenho muita tinta, gosto muito de pintar. Estou pintando uma tela de 1 por 1 e meio e uma cabeceira de cama. Costura também, muita costura.* (EF10)

A procura por suporte emocional, como na terapia com psicóloga, permitiu melhor compreensão sobre a doença e as necessidades. Evidenciou-se ainda que o uso do dispositivo móvel é uma forma de enfrentamento e distração. Esse processo facilita a aceitação da doença e a reorganização interna, contribuindo para a busca de sentido em meio à incerteza.

*A terapia com a psicóloga é um cuidado que tenho agora também* [...] *as coisas vão vindo e tu vai entendendo. Tu entendes que tu precisa e está me fazendo muito bem, estou gostando.* (EF10)

*Eu fico no celular, dou uma volta no Facebook^®^ para ver quem está de aniversário. Eu faço tudo pelo celular.* (EF12)

A manutenção de atividades cotidianas e o cuidado com a aparência surgiram como formas de reafirmar a identidade pessoal e minimizar os impactos emocionais da doença. Eles refletem um esforço por parte dos entrevistados para preservar a sensação de normalidade e controle diante das incertezas provocadas pelo câncer.

*Eu vou ao trabalho, vou na academia, eu bebo socialmente. Tipo, eu saio, caminho, eu sou muito vaidosa. Eu sempre faço a manutenção das unhas em gel, cílios, até hoje.* (EF2)

*Eu não gostava muito de me olhar no espelho antes, e hoje o que eu mais faço é me olhar no espelho. Todos os dias eu me olho no espelho e todos os dias eu agradeço por mais um dia que eu fico aqui.* (EF11)

A busca por momentos de lazer e celebração com a família emergiu como uma estratégia significativa para o enfrentamento da incerteza gerada pelo diagnóstico, mostrando-se como um mecanismo de resgate da normalidade, manutenção da identidade e reafirmação da vida.

*Após o diagnóstico, fui começando naquela luta! Como eu fazia aniversário no final do mês, eu não comentei para ninguém, eu fiquei com aquilo só para mim. Aí esperei passar meu aniversário* [...] *combinei de fazer um churrasco para reunir a família toda no meu aniversário. Eu disse: “Vamos para o camping, que é o que a gente gosta”, aí fiz! Quero bolo, quero parabéns, quero tudo! E era isso que eu queria, queria me sentir bem, queria me sentir viva, né?* (EF9)

*Gosto de sair com meu marido e passear; adoro ir para um barzinho. Gostamos de tomar uma cerveja sem álcool.* (EF14)

O contato com a natureza também foi mencionado como uma estratégia de lidar com sentimentos negativos e de encontrar a força interior. Essas práticas refletem a importância desta estratégia de enfrentamento diante da incerteza vivenciada.

[...] *eu gosto muito é de ir para o meio do mato. Lá eu me sinto bem e começo a conversar com o senhor, e então eu choro muito. Quando passa, eu volto para casa como se tivesse limpa de tudo. Eu gosto de ter o meu tempo sozinha, é onde eu me renovo. (*EF11)

[...] *eu e meu marido vamos muito para fora, que é uma coisa que a gente gosta muito. E eu caminho naqueles campos, naquelas cachoeiras, e sentar lá, aquilo ali me faz muito bem.* (EF14)

A busca por terapias complementares também foi sinalizada pelos participantes como uma estratégia de cuidado. Ademais, um deles sinalizou a utilização da cirurgia espiritual vinculada à religião como forma de enfrentar a incerteza.

*Tomo chás, chás para aumentar a imunidade, tomo chá de camomila, chá de aranto, chá de erva doce.* (EF11)

*A estratégia de cuidado que tive foi na religião, fiz cirurgia espiritual. Eu faço aplicações, tem a mãe Jurema que fala que é cirurgia.* (EF12)

### Recursos acessados pelas pessoas com câncer diante da incerteza da doença

Após o diagnóstico de câncer, os recursos acessados desempenham um papel fundamental na reorganização da incerteza. Nesta pesquisa, houve menção aos profissionais de saúde, à rede de apoio composta por familiares, amigos e vizinhos, além da religião e da fé.

As falas destacam os médicos como figuras centrais na mediação da incerteza por meio do diagnóstico e do fornecimento de informações.

*E foram os médicos mesmo que me explicaram desde o começo. Assim que eu comecei a ter suspeita, os médicos já foram passando o que era.* (EF1)

[...] *o médico falou assim direto, sabe. Pelo exame que eu fiz, já dava pra ver que era, mas só de saber e ouvir do médico, não é fácil, porque o meu câncer, a doutora não sabe bem ao certo, mas está na coluna.* (EF3)

Outro recurso mencionado foi o suporte familiar, sendo um dos mais acessados para enfrentar o diagnóstico e o tratamento do câncer. As fontes de apoio incluem familiares, amigos e vizinhos, reforçando o papel coletivo da rede de suporte e desempenhando um papel crucial na reorganização das incertezas.

*Eu tenho uma amiga, que é muito próxima, minha comadre também, então é com ela que eu consigo desabafar. Então, no fim, seria meu esposo e minha amiga. E eu estar com meu filho, meu filho que me dá força para seguir.* (EF3)

*A família é tudo. Eu tenho um filho que mora do lado, que são os que me cuidam mais de mim, porque moro ali. A minha nora, eu acredito que ela é meu anjo protetor, meu anjo da guarda, porque eu falei: “Graças a ela que eu estou aqui hoje, porque ela agilizou tudo”.* (EF12)

Por fim, as falas dos participantes revelaram a influência da religião, especialmente a umbanda, no enfrentamento do câncer. A comunidade religiosa emergiu como fonte de apoio emocional, prático e espiritual, auxiliando-os a lidar com os desafios do tratamento e a encontrar força para seguir em frente, emergindo como um refúgio em meio à incerteza.

*Ali* [religião umbanda] *que eu pego as minhas energias. É participando, né, nos dias que tem, recebendo uma palavra do irmão de corrente, de um guia espiritual que isso tudo vai passar, que eu não estou sozinha, que lá em cima rege um Deus, rege o médico que é o nosso pai oxalá, então é dali que eu também pego um pouco de energia para mim também.* (EF8)

*A religião evangélica é muito importante. Eu não sou uma frequentadora ativa, sabe, assim, aquela regrada. Gosto muito de ir aos domingos, mas me fez um bem enorme. Fez toda a diferença, me ajudou muito para enfrentar lá desde o início. A minha família foi muito importante, mas a igreja em si te ajuda bem mais para quem tem fé, né; fez toda a diferença.* (EF9)

A fé em uma força maior representou perspectiva de esperança e sentido de propósito, aliviando a angústia e a incerteza diante do diagnóstico de câncer:

*Eu tenho muita fé, muita fé, muita crença. Deus é a minha base.* (EF10)

*Eu acho que, se não existisse Deus na minha vida, se Cristo não existisse na minha vida, eu não sei o que teria passado. Desde o dia da cirurgia que eu fiz, depois do tratamento, eu sempre tive a certeza. Nos momentos mais difíceis, Deus me carregou no colo, e nos outros, sempre me estendeu a mão.* (EF11)

## DISCUSSÃO

O câncer, ao impor desafios significativos devido à sua natureza crônica e potencialmente letal, desperta incertezas nas pessoas, especialmente em relação ao prognóstico e à eficácia do tratamento. Essas incertezas geram sentimentos adversos que podem desestruturar emocionalmente as pessoas. Nesse contexto, compreender as estratégias de cuidado adotadas por pessoas com câncer diante da incerteza da doença se torna essencial, considerando a complexidade emocional e social associada a essa condição^(7,9,16)^.

As estratégias de cuidado no enfrentamento do câncer configuram formas de *coping,* ou seja, estratégias cognitivas e comportamentais utilizadas para restaurar o equilíbrio diante de situações imprevisíveis^(6)^. No presente estudo, foi identificado o uso da arte durante o tratamento, desempenhando um papel fundamental na gestão da incerteza que permeia esse momento, proporcionando às pessoas uma forma de expressar sentimentos e elaborar emoções^(7,9)^. Além disso, essas práticas artísticas permitem ressignificar a experiência vivida, transformando a vivência do diagnóstico e tratamento em algo que pode ser compreendido e enfrentado de maneira mais positiva.

Estudo realizado com adultos com doenças onco-hematológicas em hospital público universitário de Goiás destacou que a arteterapia é uma forma eficaz de expressão pessoal, contribuindo para o fortalecimento das capacidades individuais, o enfrentamento da doença e o acesso a emoções profundas^(17)^. A arteterapia pode reduzir a ansiedade e a depressão, e melhorar a qualidade de vida, sendo uma abordagem terapêutica eficaz no cuidado de pessoas com câncer^(18)^.

A terapia psicológica se destacou como estratégia de enfrentamento por favorecer a aceitação da doença, a reorganização emocional e a busca de sentido em meio à incerteza. Tal achado converge com pesquisa^(19)^ que argumenta sobre o suporte terapêutico psicológico ser tão essencial quanto os demais tratamentos oncológicos. Isso porque as intervenções psicológicas oferecem suporte emocional e ajudam a lidar com os desafios da doença e do tratamento, pois possibilitam lidar com os desafios e repercussões emocionais do câncer, abordando também questões existenciais sobre viver e morrer, promovendo enfrentamento adaptativo e humanizado^(19)^.

O uso de dispositivos móveis também foi referido como estratégia de enfrentamento e meio de distração e apoio durante o tratamento. Estudo realizado em Maringá^(20)^ evidenciou que o acesso à informação qualificada por meio dessas tecnologias favorece o autocuidado, o empoderamento e a autonomia na tomada de decisões, reduzindo incertezas e fortalecendo fatores de proteção. Assim, intervenções baseadas em *smartphones* se mostram eficazes para o desenvolvimento de competências relacionadas ao autocuidado e à decisão compartilhada.

Tanto as estratégias de suporte emocional quanto o uso de dispositivos móveis desempenham papéis complementares no enfrentamento do câncer. A terapia psicológica se mostra essencial para o acolhimento e a reorganização interna das pessoas com a doença. Por outro lado, os dispositivos móveis, quando utilizados como ferramentas educativas e de distração, promovem o autocuidado, fortalecem a autonomia e reduzem as incertezas enfrentadas durante o tratamento^(7,9)^.

Alguns participantes deste estudo adotaram estratégias de cuidado com o próprio corpo para lidar com as incertezas causadas pelo câncer, como manter a rotina diária e cuidar da aparência. Essas ações refletem um esforço para preservar a sensação de normalidade, fortalecer a autoestima, e enfrentar a doença com mais controle sobre o corpo e a vida. De forma semelhante, estudo^(21)^ evidenciou que os efeitos colaterais do tratamento do câncer podem impactar negativamente a qualidade de vida das mulheres. Em relação à imagem corporal, muitas participantes relataram não se sentir atraentes ou satisfeitas com sua aparência, especialmente em casos de câncer de mama e colo do útero. Isso está ligado aos estereótipos que sexualizam e valorizam partes específicas do corpo feminino, como seios e cabelos, tornando as mudanças físicas causadas pelo tratamento uma fonte de insatisfação. Esses aspectos reforçam a necessidade de que profissionais de saúde abordem questões de sexualidade nas consultas, considerando seu papel central na autoestima.

Uma participante, em particular, relatou uma transformação significativa em sua relação com o corpo e com a vida, evidenciando um processo de adaptação emocional diante das incertezas causadas pelo câncer. A mudança na forma como passou a se olhar no espelho reflete uma ressignificação do próprio corpo, promovendo uma nova percepção de gratidão e aceitação diante da vulnerabilidade imposta pela doença. Da mesma forma, pesquisa^(22)^ com mulheres com câncer de mama após a mastectomia demonstrou que a imagem corporal é moldada ao longo da vida por experiências, transições e eventos marcantes, como doenças que afetam a relação com o corpo e o entorno. No tratamento oncológico, as mudanças físicas, emocionais e sociais podem alterar a autoimagem e impactar negativamente a vivência da sexualidade. Todavia, após o processo de tratamento e a finalização deste processo, a beleza assume um novo significado, valorizando o corpo reconstruído de dentro para fora. Superar o câncer passa a simbolizar a força e a vitória de uma mulher que lutou e venceu, desafiando a imagem de fragilidade associada à doença e ressignificando-a como símbolo de beleza e conquista.

Essas estratégias evidenciam o papel do autocuidado e da manutenção de hábitos cotidianos no enfrentamento da doença, permitindo que ambas mantenham sua identidade e um senso de controle sobre suas vidas em meio ao contexto desafiador que estão vivenciando. Essas experiências não apenas identificam um processo de adaptação emocional, mas também a tentativa de lidar com as incertezas provocadas pela doença.

Como identificado neste estudo, a busca por momentos de lazer, convivência com a família e contemplação da natureza se torna uma forma de recuperar o controle e a sensação de normalidade após o diagnóstico. A procura pelo bem-estar emocional, felicidade e conexão, mesmo diante da incerteza da doença, aparece como uma estratégia para lidar com sentimentos negativos e encontrar força interior^(7,9)^.

Achados semelhantes foram identificados em pesquisa^(23)^ na área da oncologia, que revelou significados diversos para o lazer. Entre as atividades mais citadas como representativas de lazer, estão passeios, viagens, encontros familiares e apreciação da natureza. O lazer, portanto, possui um significado individualizado, variando de pessoa para pessoa. Essas práticas estão diretamente ligadas à satisfação das necessidades pessoais e, acima de tudo, ao desenvolvimento de habilidades e ao aumento do bem-estar de cada pessoa. Essas práticas refletem a importância dessa abordagem como uma forma de enfrentamento diante da situação vivenciada.

A procura por terapias complementares, identificada entre os participantes desta pesquisa, teve como objetivo cuidar do corpo e controlar os efeitos adversos dos tratamentos, incluindo o uso de plantas medicinais e a adesão a práticas religiosas. Essas abordagens evidenciam como a religiosidade oferece conforto e esperança, auxiliando no enfrentamento das incertezas do processo de adoecimento^(7,9)^.

Nessa direção, destaca-se estudo sobre as práticas de cuidado realizadas com participantes que convivem com o câncer na cidade de Pelotas, o qual destacou que o modelo biomédico, por si só, não atende a todas as necessidades das pessoas, pois cada uma atribui diferentes significados à experiência do adoecimento. Dessa forma, muitos recorrem às práticas além do tratamento convencional, como o uso de chás, mudanças alimentares, benzeduras, Reiki, simpatias e até cirurgias astrais, em busca de alívio ou cura. As abordagens de cuidado variaram conforme as necessidades de cada participante que, embora não convencionais, foram percebidas como eficazes. Assim, essas terapias complementares podem ser adotadas em conjunto com o tratamento tradicional, visando minimizar efeitos colaterais e melhorar a qualidade de vida^(24)^. A integração de terapias complementares com a medicina tradicional é vista como uma forma de lidar com as incertezas associadas ao processo de adoecimento e recuperação^(7,9)^.

Diante da complexidade dos cuidados exigidos pela pessoa com doença oncológica, é essencial garantir a continuidade da assistência além do ambiente hospitalar ou ambulatorial, permitindo que ele se sinta acolhido e seguro para enfrentar todas as fases do tratamento. Entre os profissionais da equipe multidisciplinar em saúde, a enfermagem desempenha um papel fundamental no suporte ao enfrentamento do câncer, empregando estratégias que visam minimizar o desconforto físico e emocional, proporcionando uma melhor qualidade de vida^(25)^.

Em consonância com o exposto, estudo sobre a função do enfermeiro no cuidado em oncologia refere que o enfermeiro é reconhecido por sua capacidade de integrar a equipe de saúde, a pessoa doente e seus familiares. Com uma comunicação assertiva e objetiva, ele consegue orientar, esclarecer, reforçar e validar as informações recebidas de diferentes fontes. O empoderamento proporcionado pelos profissionais de saúde às pessoas com doença oncológica, por meio de informações adequadas, favorece o autoconhecimento e a participação ativa no planejamento do tratamento, resultando em maior autoconfiança e melhoria na qualidade de vida^(26)^.

A compreensão desses processos de incerteza e *coping* é essencial para a enfermagem, que ocupa posição estratégica no apoio à adaptação da pessoa com doença oncológica. O cuidado de enfermagem deve, portanto, incorporar abordagens que favoreçam a construção de significado e o fortalecimento das estratégias de enfrentamento, tais como as evidenciadas nesta pesquisa. Diante disso, torna-se essencial implementar essas estratégias de cuidado na assistência de enfermagem nos ambulatórios de quimioterapia e radioterapia. O enfermeiro assume um papel central na humanização da assistência, garantindo um suporte integral e individualizado.

### Limitações do estudo

Como limitações do estudo, destaca-se o fato de apenas uma instituição ter sido cenário da pesquisa, o que demanda a relativização dos resultados. Além disso, nas primeiras entrevistas, foi necessário um processo de adaptação à abordagem qualitativa, especialmente na reformulação das perguntas, para que pudessem captar, de forma mais eficaz, os elementos subjetivos e experienciados pelos participantes.

### Contribuições para a área da enfermagem, saúde ou políticas públicas

Esta pesquisa contribui para a área do conhecimento, ao ampliar a compreensão sobre as vivências das pessoas com câncer diante da incerteza da doença, oferecendo subsídios para o aprimoramento das práticas assistenciais. Os achados podem fundamentar ações voltadas à implementação dessas estratégias de cuidado na assistência de enfermagem que ajudem as pessoas na incerteza da doença, reforçando a importância de um cuidado humanizado e centrado na pessoa no contexto oncológico. Além disso, os resultados apresentam implicações diretas para a formação de profissionais de enfermagem, ao fornecer ferramentas e estratégias que podem ser incorporadas na educação continuada e permanente, visando ao desenvolvimento de competências em cuidado humanizado, comunicação terapêutica e abordagem da incerteza. No âmbito da pesquisa, estimulam investigações futuras sobre intervenções que potencializem a centralidade do paciente e a prática baseada em evidências. Por fim, reforçam a importância de integrar essas estratégias à prática assistencial, promovendo cuidados mais seguros, efetivos e centrados nas necessidades individuais da pessoa com doença oncológica.

## CONSIDERAÇÕES FINAIS

Esta pesquisa revelou que as pessoas com câncer adotam, como estratégias de cuidado para lidar com a incerteza da doença, práticas artísticas, suporte psicológico, uso de tecnologia, manutenção das atividades cotidianas, cuidado com a aparência, momentos de lazer e celebração com a família, contato com a natureza, e terapias complementares. Essas estratégias demonstram a capacidade de auto-organização e o pensamento probabilístico da pessoa acometida com câncer, evidenciando o impacto positivo dessas práticas no enfrentamento da incerteza e no fortalecimento de sua autonomia. Entre os recursos acessados pelos participantes, estavam os profissionais de saúde, a rede de apoio familiar e de amigos, além da fé e da religião. Quanto às práticas religiosas, a umbanda foi a mais citada, destacando-se como fonte de apoio e conforto.

Sugere-se que pesquisas futuras investiguem a adesão a essas estratégias desde o início do tratamento, acompanhando a evolução das pessoas com doença oncológica que não utilizam tais estratégias e comparando com aquelas que as implementam ao longo do tempo.

## Data Availability

Os dados de pesquisa estão disponíveis no corpo do artigo.
